# Cost effectiveness and budgetary impact of the Boston University approach to Psychiatric Rehabilitation for societal participation in people with severe mental illness: a randomised controlled trial protocol

**DOI:** 10.1186/s12888-015-0593-8

**Published:** 2015-09-15

**Authors:** Sarita A. Sanches, Wilma E. Swildens, Jooske T. van Busschbach, A. Dennis Stant, Talitha L. Feenstra, Jaap van Weeghel

**Affiliations:** 1 Altrecht Mental Health Care, Lange Nieuwstraat 119, 3512 PG Utrecht, The Netherlands; 2 Tranzo Scientific Center for Care and Welfare, Tilburg University, Tilburg School of Social and Behavioral Sciences, PO Box 90153, 5000 LE Tilburg, The Netherlands; 3 University Center for Psychiatry, University of Groningen, University Medical Center Groningen, P.O. Box 30001, 9700 RB Groningen, The Netherlands; 4 Department of Movement and Education, Windesheim University of Applied Sciences, Postbus 10090, 8000 GB Zwolle, The Netherlands; 5 Zovon, Walhofstraat 28, 7522 BL Enschede, The Netherlands; 6 Department of Epidemiology, University of Groningen, University Medical Center Groningen, P.O. Box 30001, 9700 RB Groningen, The Netherlands; 7 Phrenos Centre of Expertise, PO Box 1203, 3500 BE Utrecht, The Netherlands; 8 Parnassia Group, Dijk en Duin Mental Health Center, PO Box 305, 1900 AH Castricum, The Netherlands

## Abstract

**Background:**

People with Severe Mental Illness (SMI) frequently experience problems with regard to societal participation (i.e. work, education and daily activities outside the home), and require professional support in this area. The Boston University approach to Psychiatric Rehabilitation (BPR) is a comprehensive methodology that can offer this type of support. To date, several Randomised Controlled Trials (RCT’s) investigating the effectiveness of BPR have yielded positive outcomes with regard to societal participation. However, information about the cost-effectiveness and budgetary impact of the methodology, which may be important for broader dissemination of the approach, is lacking. BPR may be more cost effective than Care As Usual (CAU) because an increase in participation and independence may reduce the costs to society. Therefore, the aim of this study is to investigate, from a societal perspective, the cost-effectiveness of BPR for people with SMI who wish to increase their societal participation. In addition, the budget impact of implementing BPR in the Dutch healthcare setting will be assessed by means of a budget impact analysis (BIA) after completion of the trial.

**Methods:**

In a multisite RCT, 225 adults (18–64 years of age) with SMI will be randomly allocated to the experimental (BPR) or the control condition (CAU). Additionally, a pilot study will be conducted with a group of 25 patients with severe and enduring eating disorders. All participants will be offered support aimed at personal rehabilitation goals, and will be monitored over a period of a year. Outcomes will be measured at baseline, and at 6 and 12 months after enrolment. Based on trial results, further analyses will be performed to assess cost-effectiveness and the budgetary impact of implementation scenarios.

**Discussion:**

The trial results will provide insight into the cost-effectiveness of BPR in supporting people with SMI who would like to increase their level of societal participation. These results can be used to make decisions about further implementation of the method. Also, assessing budgetary impact will facilitate policymaking. The large sample size, geographic coverage and heterogeneity of the study group will ensure reliable generalisation of the study results.

**Trial registration:**

Current Controlled Trials: ISRCTN88987322. Registered 13 May 2014.

## Background

High unemployment rates and general difficulty in undertaking activities are common among people with Severe Mental Illness (SMI) around the world [1–11]. The needs of these people comprise access to a broad array of services, which are not limited to clinical and humanitarian needs, but also include rehabilitation requirements such as support with societal participation, which is defined as engaging in meaningful daily activities [12, 13].

**Fig. 1:**
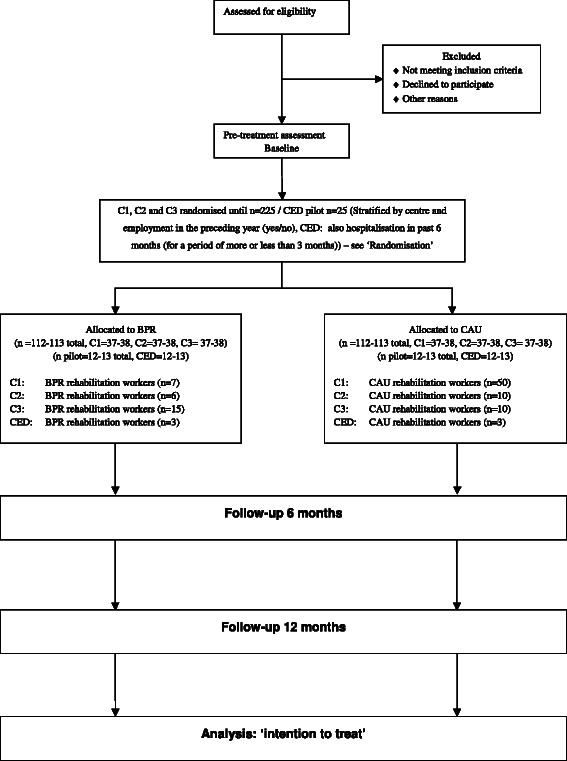
Consort diagram. Abbreviations: C1 - Centre1, C2 - Centre2, C3 - Centre3, CED – Centre for Eating Disorders, BPR – Boston Psychiatric Rehabilitation, CAU – Care As Usual

Meaningful daily activities can be described as activities that fulfil a goal or purpose that is personally and/or culturally important, and are positively correlated with quality of life [14–16]. An example of such an activity is regular employment, which is associated with higher self-esteem, fewer psychiatric symptoms, general wellbeing and higher quality of life [17–19]. When it comes to engaging in meaningful daily activities, people with SMI experience environmental obstacles such as lack of opportunities and support or negative images of the clients’ potential on the part of possible employers and others in their environments. They also have to cope with personal obstacles such as self-stigmatisation, psychiatric symptoms and lack of social skills, employment skills and emotional stability. A combination of these barriers, which often reinforce each other, can lead to motivational problems and fear of failure [20]. As a result, more people with SMI are unemployed and undertake fewer activities outside the home than may be explained by the seriousness of their psychiatric symptoms. Support in the area of societal participation is therefore vital.

Over the years, various methods have been developed to offer support in engaging in work and other meaningful activities. However, only a few of these have been proven effective. An example of a well-documented, evidence-based approach to supporting the employment needs of patients with SMI is the Individual Placement and Support model of supported employment (IPS) [21–23].

Whereas IPS focuses mainly on regular paid employment, the Boston University approach to Psychiatric Rehabilitation (BPR) has a broader scope and also focuses on other forms of meaningful activities, housing, education, social contacts, and of course, work (regular and sheltered employment as well as voluntary work). The mission of BPR is to “help persons with psychiatric disabilities increase their ability to function successfully and be satisfied in the environment of their choice with the least amount of ongoing professional intervention” [24].

In recent years, a number of RCTs have been performed which investigate the effectiveness of BPR and results are partially positive. In the United States, Rogers et al. [25] investigated the effectiveness of Psychiatric Vocational rehabilitation (BPR in the vocational context) but found no differences in vocational or clinical outcomes compared to a control condition. However, the researchers made considerable enhancements to the control condition, which may have diminished the contrast between the experimental and control condition. Positive results were found by Shern et al. [26], also in the United States, who focused on street-dwelling individuals with psychiatric disabilities. They found that the experimental group experienced fewer psychiatric symptoms and a better quality of life than the control group. Also, the experimental group spent significantly less time on the streets and showed increased societal participation in terms of a greater likelihood to attend a day programme than the control group. In Italy, Gigantesco et al. [27] investigated a specific structured planning and evaluation approach called VADO (in English, Skills Assessment and Definition of Goals) which is inspired by BPR. They found greater improvement in the personal and social functioning in patients treated with VADO compared to routine care. Swildens et al. [9] performed a multisite RCT in the Netherlands with 156 SMI patients in outpatient MHC, sheltered living and inpatient rehabilitation units. This study covered all rehabilitation areas: work, education, structured activities, social contacts and living independently. Patients were randomised to BPR or Care As Usual (CAU) with follow-ups after 12 and 24 months. The rate of goal attainment was substantially higher in BPR at 12 and 24 months than with CAU. Also, BPR was more effective with regard to promoting social contacts and societal participation. However, both groups improved equally with regard to quality of life, and BPR was no more successful than CAU with regard to living independently.

Despite the positive results and the subsequent recommendation of BPR in the Dutch Guidelines for Schizophrenia [28], implementation in MHC practice is still limited. The lack of insight into the cost-effectiveness structure of BPR is one of the obstacles to implementation. For instance, BPR may be more intensive than CAU and require an initial time investment from caregivers, which could later be compensated by increased independency and the empowerment of the patient. Indeed, if BPR effectively improves the societal participation of patients with SMI according to their own wishes, and patients have fewer needs, this may promote long-term improvement in functioning and have a positive effect on costs for society. A cost-effectiveness study is required to provide answers to these and other cost-related matters, and the results –if positive- may lead to broader dissemination of the approach. Therefore, in this study we aim to analyse the cost-effectiveness of BPR compared to CAU from a societal perspective. To further support policymaking regarding implementation, the trial results will be used in different implementation scenarios to assess the budget impact of implementing BPR in the Dutch healthcare setting.

### Objectives/aims

The main objective of the research is to gain insight into the effectiveness and cost-effectiveness of the Boston University approach to Psychiatric Rehabilitation (BPR) compared to Care as Usual (CAU) for patients with SMI (aged 18–64 years) who wish to increase their societal participation.

The primary outcome criterion is change in societal participation, which is defined as the presence of meaningful activities: paid work, regular voluntary work, vocational training or academic study, or full-time care of a family [9]. Secondary outcome measures are: patients’ experience of success, quality of life, psychosocial functioning, recovery, self-efficacy, health status and realising patients’ personal rehabilitation goals. Furthermore, we will conduct a budget impact analysis (BIA) to estimate the consequences of the broader implementation of BPR in the Dutch healthcare system.

The main research question is:

- Is BPR effective as well as cost-effective with regard to societal participation compared to CAU after 12 months for patients with SMI between the ages of 18 and 64 who have a wish for change in this rehabilitation area?

Secondary research questions are:

- Is BPR an effective intervention with regard to societal participation, in terms of patients’ experience of success, quality of life, psychosocial functioning, recovery, self-efficacy, health status and realising patients’ personal rehabilitation goals after 6 and 12 months for our target group?
- What are the financial consequences of broader implementation of BPR in the Dutch healthcare system?

## Methods

### Study design

A multicentre two parallel arm Randomised Controlled Trial (RCT) with repeated measures will be used to compare the cost-effectiveness of BPR with CAU in improving societal participation.

CAU encompasses various approaches commonly used to support patients with SMI. These include generic programmes for mental health nursing care, social work and vocational rehabilitation. CAU is provided by multidisciplinary MHC teams with assertive outreach elements for patients who need more intensive contact (Flexible ACT teams) and by Dutch regional institutes for Residential Care (RIRC, Dutch acronym RIBW), which support independent living in the community and offer supported housing facilities [29–32].

Participants will be randomly allocated to either the experimental group or the control group. Outcomes will be assessed at baseline, 6 and 12 months. Based on main study results as well as dropout rate and degree of adherence, scenarios for implementation will be formulated and assessed for their budgetary impact. An overview of the study design is shown in Fig. 1.

### Participating centres/setting

The study will be conducted at two regional MHC centres and a Dutch regional centre for Residential Care. In addition, a specialist team for SMI patients with long and enduring eating disorders will participate in the study.

### Participants

The study will include a heterogeneous group of 225 adult outpatients with SMI who express a wish to increase their societal participation. SMI is characterised by psychiatric disorders with a long duration of treatment and severe dysfunction. In the Netherlands, approximately 160,000 patients between 18 and 65 suffer from SMI [33]. Although most patients in this group have a history of psychosis, the definition of severe dysfunction is not limited to those with psychotic disorders [34]. Some patients have multiple diagnoses such as varying combinations of psychotic disorders, bipolar disorder, anxiety disorders, personality disorders, eating disorders and drug addiction. All patients are care recipients of the participating organisation that supports independent living in the community and offers supported housing facilities, or of FACT teams [35] at the participating regional mental health care (MHC) centres. A pilot group of 25 patients with severe and enduring eating disorders –often in combination with other severe psychiatric diagnoses- will be included in the study to add insight into the effectiveness of BPR for this group of long-term mentally ill patients. Patients with eating disorders also face problems such as entering or re-entering the labour market and/or finding meaningful ways of spending the day. These patients are often hospitalised for extreme underweight, meaning that this group will consist of outpatients as well as inpatients.

MH professionals will assess eligibility for participation in the study according to the following inclusion and exclusion criteria:

#### Inclusion criteria

Patients are eligible for participation if they:

- Have been diagnosed with Severe Mental Illness (a diagnosis according to DSM IV, long duration of service contact and functional impairment which substantially interferes with or limits one or more major life activities [36]) and/or have a severe and enduring history of Anorexia Nervosa.
- Have expressed a wish for change in societal participation (work, education, daily activities outside the home).
- Are between 18 and 64 years of age.^1^
- Are willing to participate in the study.

#### Exclusion criteria

Patients will be excluded if they:

- Have received BPR in the four months preceding the start of the trial.
- Are admitted as inpatients to a MHC centre at the start of the study (except for patients with Anorexia Nervosa).^2^
- Are legally incompetent.

### Interventions

#### Experimental group: Boston Psychiatric Rehabilitation (BPR)

Patients in the experimental group will take part in rehabilitation sessions based on BPR, which uses a methodology that helps patients achieve and retain their rehabilitation goals [24, 37, 38]. The approach consists of four phases:

- Exploring: the client is supported in discovering what he/she wants to achieve.
- Choosing: the client is supported in choosing a goal in a rehabilitation area such as work or housing.
- Getting: the client is supported in carrying out the necessary interventions to attain the goal.
- Keeping: the client is supported in keeping the attained goal.

Patients do not need to have a clearly defined idea or plan for change in order to receive BPR because an important component of BPR is helping patients explore their options. Therefore, BPR attends to the needs of patients who have been suffering from mental illness for a long time and have lost confidence in their own ability to initiate change. Participants assigned to BPR will be offered at least one session every 2 weeks with an experienced BPR rehabilitation worker. There is no predefined maximum or minimum number of sessions that must be completed. Instead, patient and BPR worker plan sessions by agreement. However, BPR workers are obliged to offer one session every 2 weeks. The total number of sessions and duration of the period in which patient and worker cooperate will be registered. The intervention will be carried out by rehabilitation workers fully trained in the methodology (social workers, MH nurses, occupational and vocational therapists) who have practised with at least four patients under the supervision of a certified expert in BPR. Each participating centre will take part by involving at least four workers experienced in BPR in the BPR condition. Each worker will have an additional caseload of four research patients a year. In all three centres, a sufficient number of professionals with BPR training are available to carry out the intervention.

Fidelity in the experimental condition will be checked by means of a BPR fidelity measure, using criteria developed by the Dutch foundation for BPR ‘Stichting Rehabilitatie ‘92’ in collaboration with the Boston University Center for Psychiatric Rehabilitation. Independent registered BPR experts will use the instrument to blindly rate all patient treatment files involved in this study. Furthermore, BPR workers will receive regular feedback and supervision.

#### Control group: Care As Usual (CAU)

Patients in the control group will receive CAU conducted according to the guidelines for FACT teams [29, 35, 39, 40] and Dutch RIRCs [31]. These guidelines form a general outline of the philosophy of care for the target groups and the way care is delivered in daily practice. The FACT model, which was developed in The Netherlands [35], contains all elements of the internationally well-known Assertive Community Treatment (ACT) model [41], but FACT differs from ACT in that a FACT team also targets patients who function in a more or less stable manner. These multidisciplinary teams provide medical, psychiatric, social, financial and vocational support, which can be intensified according to the patients’ needs. Detailed information about the Dutch FACT model is described in the English version of the FACT Manual [42].

In the Netherlands, supported housing and supported independent living in the community for patients with SMI is provided by 21 RIRCs. RIRC’s are primarily concerned with patient’s daily activities but also offer some support with regard to participation in society [30, 32]. However, structural support with rehabilitation is often lacking.

In line with the experimental group, participants assigned to the control group will also be offered at least one session every 2 weeks targeting a wish for societal participation, with no predefined maximum or minimum number of sessions that must be completed. Patient and CAU worker plan sessions by agreement and the total number of sessions and duration of their cooperation will be registered.

Although MH professionals working according to FACT or RIRC guidelines (social workers, MH nurses, occupational therapists or employment specialists) may be familiar with rehabilitation, they have not received specific training in BPR. They offer additional support to patients in clarifying and achieving their rehabilitation goals on the basis of generic MHC models: mental health nursing care, social work and vocational rehabilitation programmes. All CAU workers have at least 1 year of experience with supporting SMI patients. Each participating centre will provide at least three experienced rehabilitation workers to the CAU condition and each worker will have an additional caseload of at most 4 to 5 research patients a year. In all three centres, a sufficient number of professionals without BPR training are available to carry out the intervention. Because of the generic nature of CAU, fidelity will not be measured in this condition. However, CAU workers will receive regular supervision according to the FACT and RIRC guidelines to ensure a high level of care.

#### Contamination between BPR and CAU

Because workers from BPR and CAU conditions work in the same team environment and come in contact with each other, some contamination between conditions is unavoidable. However, BPR is a complex method that cannot be properly applied without extensive training and ongoing methodological supervision. Moreover, our focus is on the possible added value of specific rehabilitation techniques and not solely on rehabilitation attitude, for example. We therefore believe that the contrast between conditions will be great enough to detect possible differences.

## Measures

All measures and related domains are summarised in Table 1. All instruments have been designed to be sensitive enough to measure change in patients with SMI. A mixture of self-reporting instruments and assessment by MH professionals is used, which is generally recommended for research with SMI patients [43].

**Table 1:** Overview of outcome and other measures

Domain	Measurement
Primary outcome measures	
Societal participation in the past 6 months	Subscale Employment/Occupation of the Birchwood Social Functioning Scale
Current level of societal participation	Dutch National Societal Participation Ladder
Total hours of societal participation	Birchwood Social Functioning Scale and Health Consumption Questionnaire
Secondary outcome measures	
Patients’ psychosocial functioning from their own perspective	Birchwood Social Functioning Scale
Patients’ psychosocial functioning from the MHC team’s perspective	Activity and Participation Scale
Patients’ experience of goal attainment (yes/no)	Structured interview
Quality of Life	Manchester Quality of Life Schedule
Recovery	Recovery Assessment Scale
Self-efficacy	General Self Efficacy Scale
Cost-effectiveness outcome measures	
Generic health Status	12-item Short Form
Medical and Nonmedical costs	Health Consumption Questionnaire
Other outcome measures	
Sociodemographics	Structured interview
Psychiatric symptoms and remission	Brief Psychiatric Rating Scale
Overall level of psychological, social and occupational functioning	Global Assessment of Functioning, Symptoms and Disabilities version
Quality of the therapeutic relationship	Helping Alliance Scale
Intervention uptake	Drop-out and non-adherence

### Primary outcome measures

1. The first primary outcome measure will be *societal participation in the past 6 months*. This is expressed in paid work, voluntary work and schooling (yes/no). This measure will be derived from the subscale Employment/Occupation of the self-report Birchwood Social Functioning Scale (SFS) [44] using a hierarchical index to dichotomise whether or not there is an increase in societal participation at 6 and/or 12 months. The SFS was constructed to measure areas of functioning that are crucial for the community maintenance of people with schizophrenia (i.e. psychosocial functioning). The SFS focuses on fundamental characteristics of societal functioning and consists of seven subscales:

1) Social engagement/withdrawal, 2) Interpersonal behaviour, 3) Pro-social activities, 4) Recreation, 5) Independence-competence, 6) Independence-performance, 7) Employment/occupation.

The SFS proved a reliable and valid instrument for measuring psychosocial functioning with a high level of internal consistency (Full scale α = 0, 80, Factor loadings >0, 70).

2. The second primary outcome is *current level of societal participation,* which will be measured using the Dutch National Societal Participation Ladder [45]. The Dutch National Societal Participation Ladder was developed for the Social Support Act (Dutch: WMO) and establishes the level of societal participation in six steps:

1 = severe social isolation, 2 = social contacts outside the home, 3 = participation in organised activities, 4 = having an unpaid job, 5 = having a paid job with support, 6 = having a paid job.

A flowchart is used to determine the right step. The bottom two steps are considered a low level of societal participation. The instrument was developed for use by local governments throughout the Netherlands and was implemented in 2008. In 2013, the instrument was used by 73 % of social services [46]. Findings from a pilot study in 2009 [45] and an evaluation study in 2011 [47], indicated that the definitions of the steps are good, the instrument leaves little scope for personal interpretation and is short, clear and easy to work with. The Dutch National Societal Participation Ladder will be further validated with the subscale Employment/Occupation of the self-report Birchwood Social Functioning Scale (SFS) [44].

3. The final primary outcome measure is *total hours of societal participation.* This will be derived from the self-report Birchwood Social Functioning Scale (SFS) [44] and from a detailed questionnaire regarding Medical and Non-Medical costs based in part on the Tic-P by Hakkaart et al. [48] (see ‘Medical Costs’ for more information).

### Secondary outcome measures

Secondary outcome measures will be:

1. *Patients’ psychosocial functioning from their own perspective* is measured by the Birchwood Social Functioning Scale (for psychometric information see primary outcome measures) [44].
2. *Patients’ psychosocial functioning from the MHC team’s perspective* is measured with the Activity and Participation Scale (perspective of MH professional) [49]. The Activity and Participation Scale captures the level of participation and activity on seven dimensions:

1) Internal social integration, 2) Basal independence, 3) Use of Media, 4) Contacts with persons outside the MHC centre/living community without leaving the centre grounds.

5) Leaving the centre grounds, 6) Potential social skills, 7) Hostility to others.

The Activity and Participation Scale can be used with outpatients as well as inpatients and has good psychometric properties (α = 0.88). Factor analysis showed one factor: ‘Level of activities and participation’.

3. *Patients’ subjective experience of goal attainment* (yes/no) in achieving societal participation goals in the areas addressed, such as work and schooling. For this purpose we use a structured interview devised for previous studies [9, 50, 51].
4. *Quality of life* is measured by the Manchester Quality of life Schedule/MANSA [52]. The MANSA is specifically constructed to measure quality of life in patients with psychiatric problems and focuses on satisfaction with life as a whole. The MANSA has been translated into Dutch by Van Nieuwenhuizen et al. [53]. Twelve subjective items are answered on a 7-point Likert scale ranging from 1 = couldn’t be worse to 7 = couldn’t be better and four objective items are answered with yes/no. MANSA shows adequate internal consistency (α = 0.74; [41], α = 0.81; [54]) and is highly correlated with satisfaction ratings on the Lancashire Quality of Life Profile (LQLP: *r* = > 0.82; [52]).
5. *Recovery* is measured with the Recovery Assessment Scale [55]. The Recovery Assessment Scale (RAS) is constructed to measure recovery from serious psychiatric illnesses. The RAS consists of a grand score and five separate factors:

1) Personal confidence and hope, 2) Willingness to ask for help, 3) Goal and success orientation, 4) Reliance on others, 5) No domination by symptoms.

RAS has 41 items and is answered on a 5-point Likert Scale ranging from 1 = strongly disagree to 7 = strongly agree. Psychometric properties have been investigated by Corrigan [55]. Cronbach’s alpha for the five factors ranges from 0.74 to 0.87. Convergent validity for the five factors ranges from moderate (R^2^ = 27.7 %) to fairly high (R^2^ = 68.9 %).

6. *Self-efficacy* is measured with the General Self Efficacy Scale (GSES) [56]. This scale consists of 10 items to measure Self-efficacy on a 4-point Likert Scale ranging from 1 = not at all true to 4 = exactly true. Perceived self-efficacy is the belief in one’s competence to tackle difficult or novel tasks and to cope with adversity in specific demanding situations. The GSES has good psychometric properties: Cronbach’s alpha ranges from 0.79 to 0.90 in various study samples and self-efficacy is positively correlated with Quality of Life [57].

The following secondary outcome measures are specifically used for the cost effectiveness study.

7. *Generic health status* is measured with the 12-item Short Form Health Survey (SF-12) [58]. The SF-12 is a short and efficient instrument to measure functional health and wellbeing from the patient’s point of view. In particular, the subjective evaluation of one’s physical, psychological and social functioning. It is a shortened version of the well-known SF-36 and covers the same 8 dimensions, with one or two questions per domain:

1) Physical functioning, 2) Role-Physical, 3) Bodily Pain, 4) General Health, 5) Vitality, 6) Social Functioning, 7) Role-Emotional, 8) Mental Health.

In our study, we will use the 4-week recall version. SF-12 shows good test-retest reliability with coefficients ranging from 0.76 to 0.89 [59]. Also, validity is good with Relative validity coefficients (RV) ranging from 0.63 to 0.92. Gandek et al. [60] tested the psychometric properties of SF-12 in nine European countries and found substantial correlations between the summary measures scored from SF-36 and SF-12 Health Surveys. Also, Jenkinson [61] found SF-12 to have the same capability of measuring change in health status over time as the SF-36. We will use the methodology developed by Brazier and Roberts [62] to estimate preferences for health states based on specific items (often referred to as the SF6D) from the administered SF12. These preferences will subsequently be used to derive Quality Adjusted Life-Years (QALYs), which is a commonly used outcome in economic evaluations [63].

8. *Medical costs* will include costs of the interventions (BPR and CAU), inpatient and outpatient mental health care, general health care, costs of day activity institutions and medication use. Healthcare consumption will be registered with a detailed questionnaire administered to the respondents at each of the measurements. This questionnaire was adapted to the specific context of the current study, and partially based on the Tic-P by Hakkaart et al. [48]. Unit prices will largely be based on Dutch standard prices in order to facilitate comparisons with other economic evaluations [64]. Apart from medical costs, non-medical costs will be assessed as well.
9. *Non-medical costs* will be assessed with the same questionnaire, especially costs of informal care and costs related to productivity losses.

### Other measures

The following measures include data on possible confounding and/or mediating variables.

1. *Sociodemographic information* will be gathered by means of a structured interview scheme at the start of the study and checks will be performed in the interviews that follow at 6 and 12 months.
2. *Psychiatric symptoms and remission* are measured with the Brief Psychiatric Rating Scale [65–67] in order to control for large changes in symptomatic functioning. The BPRS was developed as a quick and efficient method to describe and evaluate change in psychiatric symptoms. We use the 24-item version which measures the following symptoms: 1) Somatic concern, 2) Anxiety, 3) Depression, 4) Suicidality, 5) Guilt, 6) Hostility, 7) Elevated Mood, 8) Grandiosity, 9) Suspiciousness, 10) Hallucinations, 11) Unusual thought content, 12) Bizarre behaviour, 13) Self-neglect, 14) Disorientation, 15) Conceptual disorganisation, 16) Blunted affect, 17) Emotional withdrawal, 18) Motor retardation, 19) Tension, 20) Uncooperativeness, 21) Excitement, 22) Distractibility, 23) Motor hyperactivity, 24) Mannerisms and posturing. Items are answered on a 7-point Likert Scale. Every scoring option has a short description of behaviours that must be observed for that particular score. The psychometric properties of the BPRS have often been investigated and BPRS has proven to be a reliable and valid instrument [68, 69]. Internal consistency is good with alphas >0.70.
3. *Overall level of psychological, social and occupational functioning* is measured with the Global Assessment of Functioning (GAF), symptoms and disabilities version (GAF-SD) [70]. GAF is scored on a scale ranging from 0 to 100 with higher scores reflecting fewer symptoms (GAF-S) and handicaps (GAF-D). The GAF-SD has proven to be a reliable and valid measure of psychiatric disturbance in a sample of people with SMI.
4. *Quality of the therapeutic relationship* is measured with the Helping Alliance Scale (HAS) [71]. The HAS was originally developed to measure the quality of the therapeutic relationship from the client’s point of view but an adapted version for the professional’s point of view also exists. Both versions consist of five items concerning the therapeutic relationship. Items are scored on an 11-point visual analogue scale ranging from 0 = not at all to 10 = entirely, with marked 10 mm intervals. Additionally, the client version has a sixth item asking about how patients feel immediately after seeing their MH professional which is answered with unchanged, better or worse. The professional version has two additional open items that ask about positive and negative aspects of the therapeutic relationship. So far, the HAS has not been psychometrically evaluated but has been used in several studies investigating the therapeutic relationship [71–73].
5. *Uptake of the intervention* will be assessed by gathering detailed information about drop-out and non-adherence during the trial.

### Sample size

With regard to the main outcome measure, the power analysis was based on the earlier finding that the proportion of patients engaged in meaningful activities increased by 11.5 % for BPR compared to 0 % for CAU after 1 year [9]. With this expected difference in proportion of change in both conditions, the study will have a power of 80 % (alpha = 0.05, two sided) if complete data from 2 × 90 patients is obtained. Furthermore, with regard to the economic evaluation, SF-12D is used to calculate a preference-based utility index, SF-6D. In the preceding RCT, the SF-12D [58] was not used, but scores on the WHOQOL are available [74]. For patients with rehabilitation goals in the area of societal participation, the differences between BPR and CAU after one year were 1.23 (SD 2.18). Calculating the required power of the study with this parameter, only 60 patients in each condition are needed to achieve 80 % power. Power rises to 95 % with 90 participants in each condition, which is needed for the primary outcome measure. Taking into account an average dropout rate of 20 % and a maximum dropout rate of 25 % after one year follow up, at least 225 respondents with SMI are needed for this trial. The number of patients eligible but not willing to participate will be carefully monitored to support the BIA. A pilot will be conducted for the additional 25 patients with severe and persistent eating disorders who will also participate in this study, since no information is available on the differences in change that can be expected after a year with and without BPR support for this group.

### Randomisation

After written informed consent has been received, an independent investigator using a stratified block randomisation scheme will assign participants to CAU or BPR. Stratification factors are ‘centre’ and ‘employment in the preceding year (yes/no)’. The group with eating disorders has an additional stratification factor: ‘duration of hospitalisation in the 6 months prior to inclusion (less/more than 3 months)’ because this group in particular needs support with rehabilitation goals directly after hospitalisation. In order to avoid large differences between both conditions in the level of costs during the period before commencement of the study, duration of hospitalisation will be added as an extra stratification factor for this group. Random allocation to either experimental group or control group will take place immediately after the baseline measurements have been completed.

### Blinding

Patients, MH professionals and research staff cannot be blinded to the allocated intervention after randomisation. However, interviewers who gather data will remain blind to treatment condition. Furthermore, research assistants blinded to treatment allocation will enter data, and research staff will only perform statistical analyses on final, anonymous datasets.

### Statistical analyses

#### Primary clinical analyses

The primary analyses will be performed according to the ‘intention to treat’ principle: all patients, including those who did not initiate or complete a rehabilitation process, will remain in the group as assigned. Missing items will be handled according to questionnaire instructions and standards. Furthermore, data will be collected from multiple sources (patients and workers), which allows indirect judgements to be used as proxies of missing data if needed.

To analyse results on the main outcome, the difference in percentage of patients with increased participation in both groups after 1 year will be compared. Firstly, descriptive statistics will be performed with regard to the proportions of patients with increased participation. Secondly, risk differences yielding numbers needed to treat (NNT) will be calculated using (multilevel) regression models to be adjusted for research centre and employment in the year preceding the study. Other possible confounders such as sociodemographics, psychiatric symptoms and remission, quality of the therapeutic relationship or educational level of MH professional will be added if comparison between intervention group and control group shows differences between these groups despite randomisation. In addition, sensitivity analyses will be performed on all outcomes, including only respondents who participated in at least two contacts with the rehabilitation worker and excluding patients for whom the outcome was rated indirectly because they were not available for interviews at 6 and 12 months. Secondary outcomes are (I) continuous variables indexing quality of life, psychosocial functioning, recovery and self-efficacy and (II) binary variables indexing patients’ experience of success in achieving their rehabilitation goals (yes/no). Patients’ experience of success in achieving their rehabilitation goals could also be presented as an (III) ordinal variable (yes/no/partially). Means and proportions of these variables at baseline, 6 and 12 months will be modelled using both multilevel linear and logistic regression analyses in which each person (level 2) contributes three observations (level 1), making adjustments for participating centre, and other confounders. Treatment effects will be quantified using the Time (baseline, 6 and 12 months) × Group (BPR and CAU) interaction, assessing whether change in the outcome of interest over time differs significantly between the two groups. Effects of time stratified by treatment group will be calculated by linear combination of the appropriate terms in the model using the General Linear Model (GLM) procedure in SPSS.

#### Cost-effectiveness analysis

An economic evaluation will be conducted alongside the clinical study to assess the cost-effectiveness of BPR compared to CAU in patients with SMI. The analysis will be performed from a societal perspective; costs in and outside the healthcare sector will be included. Time horizon of the study is 12 months, during which costs and health outcomes will be prospectively registered for all patients included. Costs and health outcomes will not be discounted due to the length of the current time horizon, which is in line with current guidelines on economic evaluations [63]. Univariate and multivariate sensitivity analyses will address important cost aspects, such as components of the intervention costs and hospitalisation. Uncertainty surrounding the cost-effectiveness and cost-utility ratios will be assessed by bootstrap analyses. In addition, cost-effectiveness acceptability curves will be used to inform decision-makers on the probability of the examined intervention being cost-effective.

Societal participation will be used as primary outcome measure of the cost-effectiveness analysis. Results will be expressed in terms of incremental costs per proportion of increase in societal participation gained. This outcome is highly relevant for patients with a history of SMI, but also closely relates to recent policy decisions on structural changes in the MHC sector (promoting de-institutionalisation and community care at lower costs). Furthermore, a cost-utility analysis will be conducted with the QALY (Quality Adjusted Life Years) as primary outcome measure. In order to estimate QALYs, utility scores will be derived from the SF-6D [62]. The SF-6D appears to have methodological advantages over other based instruments in patient populations with – mainly – psychotic disorders [75].

#### Budget impact analysis

A budget impact analysis (BIA) will be conducted to inform decision-makers about the financial consequences of the further implementation of BPR in the Dutch healthcare system. The BIA will provide insight into the total financial consequences from a societal perspective, as well as consequences for individual stakeholders in the form of an overview of net gains/costs [76]. The trial results will be extrapolated to a time horizon of up to four years and to the entire Dutch patient population concerned using health economic modelling. The model will combine the results of the cost-effectiveness analysis with epidemiological data on the targeted patient population, as well as data on programme scale and implementation. The BIA will require information about the size of the population of patients with SMI who are eligible and will participate in the intervention under study. Three scenarios will be evaluated:

1. A maximum implementation scenario assuming full participation of all eligible patients.
2. A trial scenario assuming participation rates as observed in the study.
3. A real world scenario, using larger non-participation, preferably based on previous experience with the introduction of FACT.

For all three scenarios, the 12-month results regarding societal participation will be extrapolated to results over a 48-month time horizon. This requires assumptions on how participation at 12 months will be sustained. Since specific data for the current population are lacking, we plan to use general population data concerning quit rates from various types of participation in combination with literature on similar interventions (if available). A distribution will be used to reflect uncertainty surrounding this parameter. Further modelling assumptions required concern the use of resources during months 12 to 48 and the sustainability of effects on quality of life. The BIA will be conducted and reported according to the recently updated guidelines of Sullivan et al. [77].

### Ethics committee approval

Ethical approval for this study was granted by the Medical Ethical Committee of the University Medical Center Groningen (UMCG) on 29 November 2013 (reference number: 2013/70) for all participating sites. The study is also registered with the Controlled Clinical Trials registry (ISRCTN88987322).

### Time scale

The study will be conducted from August 2013 until August 2017. Data gathering is planned from February 2014 until February 2017.

## Discussion

The aim of this study is to investigate the cost-effectiveness and budgetary impact of the Boston University approach to Psychiatric Rehabilitation (BPR) compared to Care as Usual (CAU) for people with SMI who wish to increase their societal participation. This research is highly relevant because people with SMI require support in several rehabilitation areas, in particular employment and other forms of societal participation. On average 80 % of the patients with SMI are unemployed [3–7]. This number is consistent among studies and also applies to the Dutch situation [8, 9]. Data from a large panel of Dutch patients with enduring psychiatric illnesses showed that only 16 % of the panel members had a regular paid job of 12 hours or more per week, but more than half of panel members indicated a wish to undertake more activities outside the home [10, 11]. These facts clearly illustrate a large need for support in this area, and BPR may be a successful method for providing this support.

Promoting participation in mainstream activities in the community is increasingly seen as an important MHC goal for people with SMI, and BPR could provide the much-needed support in achieving this goal.

Strength of BPR is that it focuses on all forms of meaningful activity and also provides support with more accessible activities than competitive employment, which may appeal to a broader group of SMI patients. However, a potential drawback is that goals could be set too low and patients achieve less than they are actually capable of.

With this study, we will be the first to gain insight into effectiveness, cost-effectiveness and budgetary impact of BPR, which is innovative and will make the study results of interest and applicable to MHC policy makers and healthcare funding companies. The findings of our study could potentially influence routine practice in the care of people with severe mental illness in the Netherlands as well as in other countries. The expected findings of this study will be highly relevant because the study group consists of a heterogeneous group of outpatients with SMI in different MHC settings in different parts of the Netherlands, adding to the generalisability of the results. This is important, because BPR is not specific to a particular diagnosis or group of patients. Also, this study will have a larger sample size than previous studies in the area of psychiatric rehabilitation, enabling in-depth analyses of the cost-effectiveness of BPR.

One of the design’s strengths is the active control condition, which allows for minimising the influence of attention by MH professionals and the combination of subjective and objective/clinical outcome measures. Also, treatment fidelity is measured throughout the study period, ensuring close adherence to the BPR method.

There are also some limitations associated with our study. In particular, the pragmatic approach to the study design may be a potential source of bias. For instance, centres differ in the level of BPR implementation. In some centres, BPR has been implemented for several years, whereas in others implementation is in the initial phase. In centres where BPR is almost fully implemented, the entire working context is modelled according to BPR standards. This may also have an effect on MH professionals in the control group, thus decreasing the contrast between the experimental and control conditions. On the other hand, contamination between interventions will not readily occur because BPR is too complex to adopt without proper training and ongoing supervision and the experimental condition is conducted by fully trained and experienced BPR workers.

Another limitation is the lack of standardisation of the control group. MH professionals in the control group use generic rehabilitation methods based on MH nursing care, social work and vocational programmes. This methodological diversity makes it impossible to use a fidelity measure in the control group. However, this diversity is in agreement with the reality of daily practice and workers in the control group will receive regular supervision to ensure a high quality of care.

We have mentioned the heterogeneity of our group as an advantage since it promotes generalisability of results. However, it also has the disadvantage that patients from FACT teams, regional centres for Residential Care and those with severe eating disorders in combination with other psychiatric diagnose, may have different societal and/or mental health problems and could have different participation needs. Although stratified randomisation should ensure an equal distribution in both arms, differences could impact outcomes for theses subgroups. In particular, patients with severe eating disorders may have different goals and need a different kind of support than other SMI patients. Since rehabilitation has never before been investigated among these patients, we will initially analyse this group as a separate pilot and afterwards perform sensitivity analyses on this group to investigate if adding this group to our larger sample will influence outcomes. It should be noted however, that the inclusion of a group of patients with eating disorders may also increase the relevance of this study because this group has many needs in rehabilitation areas that deserve attention, but it has not been investigated before.

Finally, patients are not blinded because this was not possible in the study design. However, to ensure data integrity, the interviewers who gather data are blinded and analyses are only performed on finalised and anonymous datasets.

If BPR proves to be a cost-effective rehabilitation method, this may lead to its further dissemination. But most importantly, it will mean that MH professionals have an evidence-based method at their disposal to fulfil the enormous need for support in societal participation and community integration among patients with SMI.

## Abbreviations

BPR: Boston University approach to Psychiatric Rehabilitation
CAU: Care As Usual
FACT: Flexible assertive community treatment
MHC: Mental health care
MH: Mental health
RIRC: regional institute for residential care
SMI: Severe Mental Illness

## Footnotes

1 Until January 1st 2014, 65 years was the retirement age in the Netherlands. After this date, retirement age is gradually being increased to 67 years. In our experience, older patients usually have different goals in societal participation – most noticeably, other than obtaining regular employment. We therefore decided to set the maximum age at 64.

2 Patients with Anorexia Nervosa are often hospitalised for extreme underweight. These patients are often young and in order to promote a smooth transition from hospitalisation to normal life, rehabilitation is started during hospitalisation. We therefore decided to include inpatients with Anorexia Nervosa. Inpatients must be in the last phase of inpatient treatment and the date of discharge must have been set.
